# Evaluation of Proinflammatory Cytokines Concentrations in Plasma, Peritoneal, and Endometrioma Fluids in Women Operated on for Ovarian Endometriosis—A Pilot Study

**DOI:** 10.3390/ijms26115117

**Published:** 2025-05-26

**Authors:** Mariusz Wójtowicz, Dariusz Zdun, Aleksander Jerzy Owczarek, Violetta Skrzypulec-Plinta, Magdalena Olszanecka-Glinianowicz

**Affiliations:** 1 Clinical Department of Gynecology and Obstetrics, Faculty of Medical Sciences in Zabrze, Medical University of Silesia in Katowice, 40-055 Katowice, Poland; mariuszwojtowicz007@onet.eu (M.W.); dariusz.zdun@gmail.com (D.Z.); 2 Health Promotion and Obesity Management Unit, Department of Pathophysiology, Faculty of Medical Sciences in Katowice, Medical University of Silesia in Katowice, 40-055 Katowice, Poland; aowczarek@sum.edu.pl; 3 Reproductive Health and Sexology Unit, Department of Women’s Health, School of Health Sciences in Katowice, Medical University of Silesia in Katowice, 40-055 Katowice, Poland; skrzypulec-plinta@o2.pl

**Keywords:** IL-6, IL-8, TNF-α, inflammation, endometriosis

## Abstract

Some studies indicate the role of TNF-α, IL-6, and IL-8 in the development of endometriosis. However, a comprehensive assessment of plasma, peritoneal, and endometrioma fluids of proinflammatory cytokine concentrations in women with ovarian endometriosis has not yet been performed. Therefore, this study aimed to analyze plasma, peritoneal, and endometrioma fluids for selected proinflammatory cytokine concentrations in women operated on for ovarian endometriosis. A retrospective study was conducted with 56 women who underwent surgery for ovarian endometriosis. Body mass, height, and waist circumference were measured, as well as BMI being calculated. Plasma, peritoneal, and endometrioma fluids’ interleukin-6 (IL-6), IL-8, and tumor necrosis factor-alpha (TNF-α) were determined by ELISA. Levels of IL-6 and TNF-α were significantly higher in endometrioma fluid compared to plasma and peritoneal fluid. In addition, levels of IL-6 and TNF-α were significantly higher in peritoneal fluid than found in plasma. Levels of IL-8 did not significantly differ between plasma and both peritoneal and endometrioma fluids, or between peritoneal and endometrioma fluids. There were also positive correlations among IL-6, IL-8, and TNF-α levels in endometrioma and peritoneal fluids (ρ = 0.29; *p* < 0.05; ρ = 0.51; *p* < 0.001; ρ = 0.52; *p* < 0.001, respectively). There were no associations between cytokine levels in plasma, peritoneal, and endometrioma fluids and endometriosis stage. Plasma IL-8 levels can be considered an emerging biomarker of severity of local inflammation related to endometrioma. Further studies are needed for understanding the role of IL-6 and TNF-α as the markers of local inflammation in endometrioma.

## 1. Introduction

Despite the relatively frequent occurrence of endometriosis, its pathogenesis is still not well known. The suggested pathological mechanisms of the development of endometriosis include uterine tissue damage or scarring related to surgery, uterine microenvironment promoting the formation of cancer, stem cells, remnant cells from menstrual blood, hormones, genes products regulating inflammation, apoptosis, invasion, angiogenesis, autophagy, and oxidative stress [1,2,3]. It has also been shown that inflammation is the one of the main mechanisms of cell proliferation and infiltration of endometriosis. Inflammation is related to the activity of macrophages, natural killer cells, T cells, and dendritic cells regulated by cytokines, prostaglandins, and chemokines [2,4,5]. Some studies demonstrated that proinflammatory cytokines such as IL-6, IL-8, and TNF-α participate in the pathogenesis of endometriosis.

A meta-analysis of 42 studies including 5414 women show significant associations between some variants of TNF-α gene polymorphisms and increased risk of endometriosis in Asian and Chinese populations [6]. It was confirmed by another meta-analysis of 34 studies which included 7521 women that additionally found associations between some variants of IL-6 gene polymorphism and increased risk of the development of endometriosis in Asian and Chinese populations [4]. The association between TNF-α gene polymorphism and risk of endometriosis has also been shown in the Greek population [5]. Furthermore, a higher expression of TNF-α was observed in endometriotic tissues compared to normal endometrium [7]. However, yet another study did not confirm the associations between some variants of TNF-α gene polymorphism and the risk of endometriosis in white women [8]. Moreover, there was significantly higher TNF-α secretion by peritoneal macrophages in patients with proven endometriosis compared to the control group [9]. In addition, TNF-α, endometrial stromal cell-derived IL-6, and monocyte chemoattractant protein (MCP-1) could stimulate peritoneal macrophages toward M2-polarization and modulate endometriosis [10]. It has also been suggested that TNF-α is responsible for peritoneal fluid-mediated enhancement of eutopic and ectopic endometrial cell proliferation in women with endometriosis [11]. One possible mechanism of TNF-α involvement is the induction of cyclooxygenase-2 overexpression in eutopic endometrium activating nuclear factor-κB [12]. Other mechanisms may be the deregulation of MMP9, proMMP9, and ICAM1 by TNF-α [13]. Still, another mechanism may be decreased progesterone receptor B/A ratio by treatment with either TNF-α or peritoneal fluids in women with advanced stage endometriosis [14].

It has been found that IL-6 may participate in the development of endometriosis by inhibiting the apoptosis of ectopic implants [15] and stimulating the migration of endometriotic implants [16]. An experimental study showed that anti-IL-6 receptor monoclonal antibody significantly suppressed the volume of endometriotic lesions compared with non-treated rats [17]. Increased levels of IL-6 were found in women with stage I-II endometriosis, with 75% sensitivity and 83.3% specificity [18]. Moreover, Socolov et al. [19] showed IL-6 above the cut-off threshold of 2 pg/mL in 71% women with endometriosis and 87% of controls; differences between the two groups was not statistically significant.

Increased peritoneal and serum IL-8 levels were shown in women with endometriosis and associations with severity of the disease, size, and number of the active lesions. It has also been found that IL-8 directly affects endometrial cell proliferation and by this mechanism, plays a role in the growth and maintenance of ectopic endometrial tissue by protecting ectopic cells against death by apoptosis [20]. The upregulated expression of IL-8 in ectopic lesions compared to eutopic endometrium samples from patients with endometriosis was demonstrated [21]. However, some studies showed no differences between IL-8 levels in serum and peritoneal fluid [22]. In contrast, other studies found increased IL-8 levels in serum and peritoneal fluid in women with stage I-II endometriosis and higher levels in endometriomas [23,24].

It has also been suggested that IL-8 stimulates endometriosis in a dose-dependent manner [25]. Moreover, a recently published study found that a long-acting anti-IL-8 antibody reduces inflammation and fibrosis in endometriosis, leading to improvement [26].

Other proinflammatory cytokines involved in the development of endometriosis include IL-1, IL-2, and IL-33. IL-1 facilitates the innervation process in endometriosis. It has also been shown that altered IL-1 expression is associated with endometriosis progression as well as pain and infertility related to the disease [27,28]. IL-2 promotes invasion and migration of endometrial cells [29,30]. IL-33 plays a role in cell invasion, migration and adhesion, neovascularization, and pain development [31,32,33].

So far, as potential markers of endometriosis glycoproteins, growth factors, miRNAs, lncRNAs, as well as proteins related to the angiogenesis process or immunology were assessed. However, no single biomarker or panel of biomarkers is sufficiently specific or sensitive enough for a diagnostic test for endometriosis [34]. The combination of CA-125 with IL-8 and TNF-α during the secretory phase of the menstrual cycle gives a sensitivity of 89.7% and specificity of 71.1% for endometriosis [35]. Moreover, the combination of CA-125, chemokine receptor (CCR) type1, mRNA, and MCP1 has a sensitivity of 92.2% and specificity of 81.6% [36]. In turn, other glycoprotein CA-19-9 levels were positively associated with the severity of endometriosis [37]. Another glycoprotein glycodelin A levels in serum and peritoneal fluid has a 91.7% sensitivity and 75% specificity for endometriosis, while IL-6 levels have 93.8% and 80%, respectively, and intercellular adhesion molecule 1 has 58.3% and 60.0%, respectively [38]. Moreover, Zinc alpha 2-glycoprotein has 46% sensitivity and 100% specificity [39].

As was described above, some studies indicate that TNF-α, IL-6, and IL-8 play a role in the pathogenesis of endometriosis. However, a comprehensive assessment of plasma, peritoneal fluid, and endometrioma TNF-α, IL-6, and IL-8 concentrations in women with ovarian endometriosis has not yet been performed.

## 2. Results

The baseline characteristics of the study group are presented in Table 1. Stages of endometriosis assessed according to revised the American Society for Reproductive Medicine classification [40] are presented in Table 2.

Levels of IL-6 and TNF-α were statistically significantly higher in endometrioma fluid than in plasma and peritoneal fluid. In addition, levels of IL-6 and TNF-α were statistically significantly higher in peritoneal fluid than in plasma. Levels of IL-8 did not statistically significantly differ among plasma and peritoneal and endometrioma fluids as well as peritoneal and endometrioma fluids (Table 3).

There were no statistically significant correlations between plasma levels of IL-6, IL-8, and TNF-α and BMI. In addition, IL-6, IL-8, and TNF-α levels in endometrioma and peritoneal fluids did not statistically significantly correlate with BMI.

A negative statistically significant correlation between IL-6 and IL-8 levels in plasma and WBC numbers was found (ρ = −0.32; *p* < 0.05 and ρ = −0.28; *p* < 0.05, respectively). There were also positive statistically significant correlations among IL-6, IL-8, and TNF-α levels in endometrioma and peritoneal fluids (ρ = 0.29; *p* < 0.05; ρ = 0.51; *p* < 0.001 and ρ = 0.52; *p* < 0.001, respectively). We also observed positive statistically significant correlations between levels of IL-8 in plasma and both peritoneal and endometrioma fluids (ρ = 0.41; *p* < 0.01 and ρ = 0.33; *p* < 0.05, respectively). In addition, there was also a positive statistically significant correlation between TNF-α and IL-8 in peritoneal fluid (ρ = 0.36; *p* < 0.05).

There were no statistically significant associations between cytokine levels in plasma, endometrioma, and peritoneal fluids and endometriosis stage.

## 3. Discussion

Endometriosis with ovarian localization named endometrioma is a specific subtype of the disease. The endometrioma is surrounded by stroma and a single layer of columnar epithelial cells and is not surrounded by a capsule. Thus, arising within it proinflammatory cytokines and ROS may diffuse into the ovarian cortex and adjacent follicles [41]. Higher levels of the interleukins (IL-1β, IL-6, IL-8, IL-15, IL-18) in follicular fluid than in serum of women with endometrioma were observed [42]. It has also been shown that these proinflammatory cytokines and ROS induce oxidative stress and fibrosis in the ovary [43].

To the best of our knowledge, this is the first study to assess levels of IL-6, IL-8, and TNF-α in plasma, endometrioma, and peritoneal fluids of women operated on for ovarian endometriosis. Our study showed significantly higher levels of IL-6 and TNF-α in endometrioma fluid compared to plasma and peritoneal fluid, as well as in peritoneal fluid compared to plasma. In addition, there were no associations between IL-6 and TNF-α levels in plasma, endometrioma, and peritoneal fluids depending on endometriosis stage. These results are in accordance with previously published data that show a higher secretion of TNF-α by peritoneal macrophages in patients with endometriosis [9]. Our observation is also similar to a study that shows a lack of association between IL-6 and TNF-α levels and stage of external genital endometriosis [44]. Moreover, another study found a lack of associations between IL-6 and TNF-α levels and both severity of endometriosis and pain symptoms [45]. However, there are also studies that found associations between IL-6 levels in peritoneal fluid and stage of endometriosis [46,47]. It has also been shown that IL-6 levels are higher already in women with stage I-II endometriosis than in women without endometriosis [18]. Additionally, Socolov et al. [19] showed no statistically significant differences between the percentage of women with IL-6 above 2 pg/mL in the groups with and without endometriosis. In addition, our study shows associations between IL-6 and TNF-α levels in peritoneal and endometrioma fluids but not between their levels in plasma and both fluids. This observation suggests that plasma IL-6 and TNF-α levels do not reflect local inflammation in endometriosis. This hypothesis is supported by a study that shows significant production of IL-1beta, IL-6, and TNF-α in endometriotic tissue [48] and another study that found both basal and stimulated synthesis of IL-6 and TNF-α in peritoneal macrophages of women with endometriosis [49]. Yet another study found that plasma IL-6 levels, but not TNF-α levels, may be useful for differentiating endometriosis from other causes of infertility [50].

As was described above, there is an association between IL-8 levels and severity of endometriosis as well as size and number of active lesions [20]. However, in our study, levels of IL-8 did not significantly differ among plasma, peritoneal, and endometrioma fluids. These are in accordance with some previously published studies [22]. It should also be noted that IL-8 levels in serum and peritoneal fluid are higher in women in endometriomas [24]. We did not observe associations between plasma and both fluid levels of IL-8 and stage of endometriosis. This is in accordance with a study that showed similar IL-8 levels in mild and severe external genital endometriosis [44]. Another study also found a lack of differences between IL-8 levels in peritoneal fluid in women with minimal/mild and moderate/severe endometriosis [51].

However, our main observation was that plasma IL-8 levels correlated with levels in both peritoneal and endometrioma fluids. Regardless of the stage of endometriosis, plasma IL-8 levels may be a marker of local inflammation related to endometriosis. Of interest, it has been shown that IL-8 may become a useful tool for differentiating ovarian endometriomas from deep infiltrating endometriosis [52]. In addition, a recently published study found an association between polymorphism of the IL-8 gene and endometriosis-related pelvic pain [53]. This suggests that IL-8 plays an important role in the development of inflammation in the course of endometriosis. Further studies performed on large groups are necessary to confirm any diagnostic role of plasma IL-8 levels in endometriosis.

The limitation of our study is the size of the study subgroups and the lack of a control group without endometriosis. Furthermore, in our study, only selected proinflammatory cytokines were analyzed. Moreover, our study was limited to only one type of endometriosis with ovarian localization.

## 4. Materials and Methods

This study aimed to analyze plasma, peritoneal, and endometrioma fluid for selected proinflammatory cytokine concentrations as potential markers of severity of endometriosis in women operated on for endometrioma.

This was a retrospective study involving 56 women who were operated on for ovarian endometriosis in the Clinical Department of Gynecology and Obstetrics, Faculty of Medical Sciences in Zabrze between 2019 and 2022. The inclusion criteria were at least 2 years primary infertility, stage III/IV ovarian endometriosis, and regular cycles. Endometriosis was diagnosed by laparoscopy and histologically confirmed and classified according to the American Society of Reproductive Medicine classification [40]. The exclusion criteria included other than ovarian localizations of endometriosis, additional extraovarian endometriosis, hormonal disturbances including thyroid dysfunction, Cushing’s syndrome, type 1 and 2 diabetes, smoking, alcohol abuse, changes in body mass during the last 3-month period, any pharmacological therapy, acute inflammation, and anything other than endometriosis with chronic inflammation. The study was conducted after obtaining informed consent of each participant, based on the study protocol, approved by the Bioethical Committee of the Medical University of Silesia (PCN/0022/KB1/136/19 dd. 3 December 2019).

Body mass, height, and waist circumference were measured, and body mass index (BMI) was calculated according to the standard formula. During the morning between 6.00 and 7.00 a.m., after an overnight fast (14 h), 15 mL venous blood samples were withdrawn. During laparoscopic operation, free peritoneal fluid from Douglas’ sinus and endometrioma fluid were collected according to recommendations of the kit manufacturers (BioVendor, Brno, Czech Republic). Serum and fluid aliquots were frozen and stored at −70 °C.

### 4.1. Laboratory Procedures

Blood morphology was assessed.

The ELISA method was used for measurements of plasma and fluids, IL-6 (BioVendor, Brno, Czech Republic), IL-8 (BioVendor, Brno, Czech Republic), and TNF-α (BioVendor, Brno, Czech Republic) with the LoQ of 0.32 pg/mL, 0.5 ng/mL, 0.13 pg/mL. Respectively, intra- and inter-assay coefficients of variations were 4.6% and 4.8% for IL-6; 4.5% and 7.1% for IL-8; and 8.5% and 9.8% for TNF-α.

### 4.2. Statistical Analysis

Statistical analysis was performed using STATISTICA 13.0 PL (TIBCO Software Inc., Palo Alto, CA, USA) and StataSE 13.0 (StataCorp LP, College Station, TX, USA). Statistical significance was set at a *p* < 0.05. All tests were two-tailed. Nominal and ordinal data were expressed as numbers and percentages. Interval data were expressed as mean ± standard deviation (normal distribution) or median (lower–upper quartiles) in the case of non-normal distribution. The distribution of variables was evaluated by the Shapiro–Wilk W test and the quantile–quantile (Q-Q) plot. The rank ANOVA was used to compare cytokine levels between plasma and endometrial/peritoneal fluid with the Tukey post hoc test. The homogeneity of variances was assessed by the Fisher–Snedecor F test. Correlations between variables were assessed with the ρ Spearman rank correlation coefficients. As this study is a pilot study, no formal sample size analysis was conducted. Yet, according to the post hoc power analysis, all analyses present results with the power of the test of at least 80%.

## 5. Conclusions

Plasma IL-8 levels can be considered an emerging biomarker of severity of local inflammation related to endometrioma. As the results concerning IL-6 and TNF-α were inconclusive, further larger studies are needed for explaining the role of IL-6 and TNF-α as the markers of local inflammation in endometrioma. It should also be noted that we did not observe in any biological material associations between study cytokine levels and the severity of the endometriosis assessed histologically in accordance with the American Society of Reproductive Medicine classification.

## Figures and Tables

**Table 1:** Baseline characteristics of study group.

N	56
Age [years]	33 ± 6
HGB [g/dL]	13.1 ± 1.1
WBC [tys/uL]	6.8 (5.6–8.1)
RBC [mln/uL]	4.4 ± 0.4
PLT [tys/uL]	258 ± 51
BMI [kg/m^2^]	22.6 ± 4.3
ESR [mm/h]	8 (4–11)
CRP [mg/L]	0.6 (0.6–1.8)

Mean ± standard deviation or median (lower quartile–upper quartile).

**Table 2:** Stages of endometriosis according to revised American Society for Reproductive Medicine classification.

Stages	N (%)
III	39 (73.6)
IV	14 (26.4)

**Table 3:** Cytokine levels in plasma, endometrioma, and peritoneal fluids.

	Plasma	Endometrioma Fluid	Peritoneal Fluid	*p*
IL-6 [pg/mL]	3.3 (1.1–12.3)	82.9 (40.7–113.2) ^#^	40.5 (5.6–89.7) ^#,^*	<0.001
IL-8 [pg/mL]	27.5 (12.2–131.2)	82.5 (45.4–113.8)	103.6 (14.0–138.7)	0.11
TNF-α [pg/mL]	0.4 (0.3–0.6)	1.4 (0.8–5.0) ^#^	0.8 (0.6–1.0) ^#,^*	<0.001

^#^ *p* < 0.001 in comparison to plasma; * *p* < 0.05 or least in comparison to endometrioma fluid; median (lower quartile–upper quartile).

## Data Availability

Data are available from the corresponding author.

## Abbreviations

The following abbreviations are used in this manuscript:

IL-6	Interleukin-6
IL-8	Interleukin-8
TNF-α	Tumor necrosis factor-alpha
BMI	Body mass index
